# Interpreting and acting upon home blood pressure readings: a qualitative study

**DOI:** 10.1186/1471-2296-14-97

**Published:** 2013-07-13

**Authors:** Konstantina Vasileiou, Julie Barnett, Terry Young

**Affiliations:** 1 Department of Information Systems & Computing, Brunel University London, Kingston Lane, Uxbridge, UB8 3PH, UK

**Keywords:** Home blood pressure monitoring, Interpretation of readings, Patient-clinician relationship, Qualitative interviews, United Kingdom

## Abstract

**Background:**

Recent guidelines recognize the importance of home blood pressure monitoring (HBPM) as an adjunct to clinical measurements. We explored how people who have purchased and use a home blood pressure (BP) monitor make sense of, and act upon, readings and how they communicate with their doctor about the practice of home monitoring.

**Methods:**

A qualitative study was designed and participants were purposively recruited from several areas in England, UK. Semi-structured in-depth interviews were conducted with 18 users of home BP monitors. The transcribed data were thematically analysed.

**Results:**

Interpretation of home BP readings is complex, and is often characterised by uncertainty. People seek to assess value normality using ‘rules of thumb’, and often aim to identify the potential causes of the readings. This is done by drawing on lay models of BP function and by contextualising the readings to personal circumstances. Based on the perceived causes of the problematic readings, actions are initiated, mostly relating to changes in daily routines. Contacting the doctor was more likely when the problematic readings persisted and could not be easily explained, or when participants did not succeed in regulating their BP through their other interventions. Most users had notified their doctor of the practice of home monitoring, but medical involvement varied, with some participants reporting disinterest or reservations by doctors.

**Conclusions:**

Involvement from doctors can help people overcome difficulties and resolve uncertainties around the interpretation of home readings, and ensure that the rules of thumb are appropriate. Home monitoring can be used to strengthen the patient-clinician relationship.

## Background

Blood pressure (BP) measurements are taken traditionally in doctors’ surgeries. Recent clinical guidelines [1-3] recognize the importance of home blood pressure monitoring (HBPM) as an adjunct to clinical measurements for the diagnosis and control of hypertension. Hypertension is a leading risk factor for disease globally [4] (e.g. cardiovascular, renal disease) affecting more than a quarter of the world’s adult population [5].

HBPM offers several clinical advantages, such as improved BP control [6-8] strong correlation with target organ damage and cardiovascular mortality [8], and the potential to overcome therapeutic inertia [7] (i.e. unchanged medication in hypertensive patients despite uncontrolled BP). Patients find HBPM an acceptable, convenient and satisfactory method of measurement and prefer it to clinical or ambulatory monitoring [9,10].

Lay people are increasingly adopting HBPM. A UK survey among a randomly selected sample showed that 9% of respondents had self-tested their BP [11]. The percentage increases considerably among hypertensive adults: 31% and 43% of the respondents in cross-sectional studies in the UK [12] and US [13] respectively reported to having measured their own BP.

Qualitative research [14-17] has highlighted the views and experiences of patients (e.g. hypertensive, stroke patients) engaging with HBPM. Patients report feeling more empowered and involved in their care [15] and more knowledgeable about the risks implicated in high BP [17]. HBPM operates to reassure and assists people to reflect on, and regulate aspects of their health management [14-16]. It is seen as an easy and straightforward technique with which most feel confident [14].

Although we are unaware of research that focuses exclusively on the interpretation of home readings, there are some hints in existing literature. Self-testing patients commonly notice variability in BP values [14-16] that may provoke a series of actions taken to regulate BP, including intentional changes in medication. BP fluctuations or unexpected readings sometimes lead to confusion and anxiety [15] and maybe disregard [16].

Previous research [15] presents a mixed picture of the doctor’s involvement, with some patients reporting disinterest by doctors when discussing the practice of HBPM. This creates reluctance to seek further medical advice, especially when home monitoring is self-initiated without a medical prompt [16]. Evidence also suggests that healthcare professionals are concerned that their workload may increase as patients become unnecessarily over-preoccupied with such measurements [18]. Interestingly, this is in contrast to patients’ views who believe that HBPM helps them visit the doctor less often and avoid ‘bothering’ him/her [14].

Building on this research, and against the background of recent guidelines, we examined in detail how people, who have purchased and use a home BP monitor, make sense of, and act upon, home readings and how home monitoring influences the patient-clinician relationship. A qualitative, interview-based study was designed to explore firstly the reasoning behind the interpretation of home readings, and secondly the way people communicate and interact with their doctor with reference to these values and the practice of HBPM.

## Methods

### Design, study population and sampling technique

A qualitative, interview-based, study was designed. The study population of interest was people who had purchased a home BP monitor for their own use. Those who had been provided with a monitor through their healthcare provider (e.g. the UK National Health Service - NHS) were thus excluded. We then purposefully selected participants to provide maximum variation [19] in relation to the health condition that triggered the device purchase.

### Setting and recruitment strategy

Prospective participants were recruited through advertisements placed at a UK University in North West London and the local community (i.e. Uxbridge). Pharmacies were also approached but adverts were not feasible to be placed at these sites. Additional participants were further recruited through the researchers’ personal networks (see Table 1).

**Table 1:** Sites of participant recruitment

**Sites**	**No. recruited**
UK University in North West London	**13**
*Academics*	*4*
*Students*	*4*
*Staff*	*2*
*Contacts of University staff outside the University*	*3*
Local community (e.g. through adverts at public library and local store)	**1**
Personal networks of researchers	**4**

Most interviewees were residents of London, and fewer were living in other areas in England (i.e. Nottingham, Milton Keynes, and Alton). People who expressed an interest in the research completed initially a short questionnaire (Additional file 1) which ensured eligibility for participation. The screening questionnaire also included questions about participants’ demographics, the self-reported health condition that prompted the device purchase (see Table 2), as well as the characteristics of the monitor (see Table 3) and its use (e.g. duration, frequency).

**Table 2:** Participants’ characteristics (N = 18)

**Characteristic**	***N***
Gender	
*Female*	7
*Male*	11
Age (years)	*M* = 54; *Median* = 55; *Min* = 23, *Max* = 93
Education	
*Degree or degree equivalent and above*	17
*Higher education to less than degree level*	1
Nationality	
*British*	14
*British/Egyptian*	1
*Non-British*	3
Employment status	
*Employed*	9
*Unemployed*	1
*Retired*	4
*Other (e.g. student, self-employed)*	4
Marital status	
*Married*	14
*Divorced*	2
*Single*	1
*Missing*	1
Self-reported health condition prompting the device purchase	
*1. Hypertension or elevated BP readings*	8
*2. High BP during pregnancy or fear of high BP during pregnancy*	4
*3. BP related problems as a result of other health conditions (e.g. cardiovascular disease, cancer) or medication*	3
*4. Hypotension*	2
*5. White coat effect*	1

**Table 3:** Device characteristics

**Characteristic**	**N**
Clinical validation	
YES	6
NO	2
‘Don’t know’	10
Type	
Automatic	17
*Arm 14*	
*Wrist 3*	
Manual (i.e. mercury)	1
Cost	
< £50	12
> £50 and < £100	6

### Data collection

Twenty-one people initially expressed an interest in the study of whom 18 were eligible. Eligible participants were invited to take part in a face-to-face, semi-structured interview which was conducted in line with the designed interview protocol (Additional file 2). Participants were informed about the purpose and the procedure of the interview and provided written consent. Interviews were carried out by KV between January and April 2012 and were recorded. At the end, participants were fully briefed and were given a £15 voucher in acknowledgement of their participation. Ethical approval was granted from the Ethics Committee of Brunel University, London.

### Data analysis

Recorded interviews were transcribed verbatim by a professional company. Scripts were subjected to thematic analysis, a suitable analytic technique for the identification of ‘repeated patterns of meaning’ [20,21]. The analysis proceeded as follows: initially there was a familiarisation process through the repeated reading of transcripts that facilitated immersion in the data. Data relevant to our research questions were noted. Next, extracts of similar meaning were assigned to developing codes, assisted by computer software (NVivo 9) [22]. No new codes were identified after the twelfth interview indicating data saturation. Semantically-related codes were then grouped together and themes and subthemes were developed. Initial coding was applied by KV, and themes and subthemes were developed, revised and refined in conjunction with JB in regular meetings.

## Results

### Participants

In total, 18 users of home BP monitors were interviewed. Seven were female and 11 male, aged between 23 and 93 years old, and all were well educated. The self-reported health conditions that triggered the device purchase were hypertension, hypotension, white coat effect, and BP-related problems as a result of other conditions or medication (Table 2). All interviewees had an automatic monitor except for one who used a manual device (Table 3).

## Analysis

The analysis is structured in two main sections; the first concerns the interpretation of readings and the actions taken as a result of this, and the second pertains to the communication of the practice of HBPM to the doctor. Quotes are identified by participants’ gender (M: male, F: female), age and unique coding number.

### A. Interpreting and acting upon readings

#### A.1. Mental models of BP function

Participants had developed certain ideas about the function of BP drawing from their experience of self-monitoring and from their general knowledge. One common observation they made was that BP is not consistent but fluctuates considerably within and across days. Dietary intake, exercise and psychological states were all considered to account for this variability.

But again, I know from my own work that you can…you know, the blood pressure is changing fairly rapidly throughout the day, so when you monitor at a different point in the day, depending what you’re doing, what you’ve been eating and what you’ve been drinking, it can all affect it. (M, 53, 1016)

The fluctuating nature of BP sometimes generated uncertainty as to whether the values genuinely represented participants’ physical condition. This led to questioning either the informational value of readings altogether or the accuracy of the monitor.

P: ….the measurements are quite ropey actually. They’re fairly inconsistent measurements, so that the… You can put it on one minute, get one reading, and then do it two minutes later and get a different reading.

I: Yeah, a different one.

*P: Yeah, so you’re not – it’s not really…there isn’tablood pressure that you’re getting. That’s part of, obviously, the blood pressure itself isn’t one thing, you know, one measurement, all the time, so it’s a bit difficult to work out at what point you are…getting it. (M, 53, 1011)*

In other instances, the uncertainty induced by BP variability made participants doubt whether they were using the device properly.

I: So do you feel pretty confident when you are using the monitor?

P: I’m not 100% confident because of those figures that came there…What are they…? Yes, I took these today. The blood pressure seems to jump about a lot. Yes, March…that’s March 6th, this morning…and two days ago, it was much lower.

I: Yeah, I can see that.

P: And I’m not sure why. (F, 93, 1012)

Having multiple measurements that would enable users to see the trend of BP over a period of time and get the average was considered by many to be more useful than simply relying on single readings.

#### A.2. Normality of BP values and personalisation

Most participants in this study showed awareness of the BP values that are considered to be normal for adult populations although these guideline values were not the only heuristic or ‘rule of thumb’ on which users relied to judge readings. A parallel, and arguably strong, heuristic was based on personalised norms of values which had been developed according to the history of users’ own readings. These individualised norms defined not only average values, but also an acceptable range of values within which they felt comfortable. It was when readings were outside this personalised comfort zone that further action was more likely to be triggered. A man with hypertension stated:

I: So how and when do you know that the readings are normal for example?

M: I was told, at the surgery, it’s about…in the 140s. So once I… if I’m hitting 160, 170, 180, then it’s very high, too high, but then it, as I say, it does come down….Em…but yes, I’m conscious now, and I’m pleased anything probably…what…below 160?

I: Below 160?

M: If I can get to below 160, 150s, then I think that’s good. Compared to 170, 180, 190, you know. (M, 62, 1018)

For a few participants designating the acceptability of readings appeared to be a difficult task. Again, the personalised norm of BP values was employed to make sense of the output. Here, the participant’s rule of thumb draws attention mainly to the upper limit of the personal range which is taken as her own critical threshold.

I do have an idea of what I think is normal for me, yes. It’s always quite hard to work that out, isn’t it? But of course, because I’ve kind of gone on this journey of having alright blood pressure, and then high blood pressure, I know, at the worst point, what my really high blood pressure was, so I kind of think anything under that is alright. I don’t think it’s necessarily straightforward to work that out, but I do have in my mind what I think is alright or not. (F, 46, 1008)

Clearly, participants were trying to find functional ways to define acceptable ranges of BP values. The guideline values were an initial broad framework for judgements, but within this, the personalised norm was deployed as a more meaningful rule of thumb, especially for those users who had a relatively consistent history of elevated or reduced readings.

#### A.3. Explaining readings: contextualisation and attribution process

The need to make sense of the home readings was mostly evident when the values were designated as problematic, that is, outside the range of values considered to be acceptable according to the two main rules of thumb. In this case, an attribution process [23] was initiated during which participants were seeking to identify the potential causes of the problematic readings.

I: What did you do [when you got problematic readings]?

P: Just eh…think of what I ate, think of what I did, mainly yes, think of why…why has it gone up… (M, 48, 1009)

Identifying the potential causes of high BP was important and operated to reassure participants as they could explain the situation and direct action accordingly. Obtaining an odd reading for which there was no ready explanation caused greater concern compared to the situation where the potential reasons could be determined.

I’ll be more concerned if I was eh…in a relaxed situation, em, sitting down and I got a very high reading for no reason at all. (M, 65, 1002)

Apart from diet and exercise, psychological states, such as stress, anxiety or surprise, and physical conditions, such as having a flu or weight changes, were also referred to as potentially affecting BP levels.

I know what my blood pressure is now, and I know what it kind of should be, and I know occasionally when it’s gone above that, and I kind of have a good idea sometimes of why it’s gone above - like if I’ve put weight on or whatever, it seems to go higher or whatever, and if I’ve lost weight, then it kind of goes down again. (F, 46, 1008)

Clearly, the interpretation of readings was often extended beyond a mere assessment of value normality. People also tried to understand their causes, not only by using the lay models of BP function, but also by contextualising the values in personal – immediate or longer-term - lifestyle parameters (e.g. exercise, diet), psychological states (e.g. stress) and parallel physical conditions (e.g. flu). The attribution process was mostly evident when the readings were perceived as problematic, since determining the causes helped users decide on relevant action.

#### A.4. Acting upon readings

The actions that participants reported to taking as a result of getting unusual readings depended on the outcome of the attribution process. However, as part of their overall risk assessment, they firstly tried to establish the consistency of the problematic values. Several users mentioned that they were taking additional readings in order to check the reliability of the first one.

If I had a high number, then I would take two or three readings. If the first one was, oops, that’s a bit high, then I would take two or three readings and then see if it was…something that I’d just done or if it was staying high. But if it comes out okay on the first reading, I don’t worry to do it again. (M, 60, 1010)

If the problematic readings persisted, then action was initiated that aimed to regulate BP. Immediate dietary changes were commonly introduced leading participants to include or avoid certain foods that were believed to affect BP. Resting was another common practice whilst more psychologically-oriented actions were also adopted to alleviate intense emotions that were considered to impact on BP levels.

I: And what did you usually do when you were getting bad readings?

P: I’d try and do more relaxation. (M, 60, 1010)

For most participants contacting the doctor to handle problematic readings appeared to be a last resort. This action was initiated when people did not succeed to regulate BP through their other interventions, and when the problematic readings persisted and could not be readily explained.

If it suddenly started to go up to 180 and 190 or something like that, I would realise something is going wrong. If I didn’t even know what it was going wrong, I would seek medical advice, you know. (M, 77, 1014)

### B. Communicating the practice of home-monitoring to the doctor

Most participants had informed their doctor that they owned and used a home BP monitor except for three who felt that their health condition was not that serious or urgent enough to be worthy of discussion or did not have the chance to do so. Among those who had notified their doctor, there were various accounts of the nature of this engagement. Some reported that their doctor did not really engage in extensive discussions or comments about HBPM and that they rather seemed to adopt a neutral or disinterested stance.

I went back quite soon after the initial diagnosis just to confirm that everything was okay. I think I mentioned that I’d purchased this, but that was it really. We didn’t really speak about it any more than that. (F, 57, 1005)

In contrast, others mentioned a high level of involvement on the part of their doctor that made them feel truly supported in their efforts to manage their condition. One participant, indeed, expressed surprise from the level of her doctor’s engagement and the fact that her home readings were noted and taken into account.

But I was surprised that you could have a do-it-yourself test and they would be quite happy just to… And in fact, they would write down what I’d told them in my notes, which I was really surprised at. (F, 46, 1008)

Support from doctor was sometimes explained by the fact that the users possessed some sort of medical knowledge as a result of their own professional expertise, which in turn was believed to make the doctor have faith in what they were doing.

But she is very happy, but she knows that we both have been involved in medical work and that, you know, so she has a degree of trust in what we say and do. (M, 77, 1014)

Lacking medical knowledge, on the other hand, was viewed to account for the perceived doctors’ distrust that had been experienced by some users. In this case, people felt that their doctor did not have confidence in their home readings due to a lack of medical background. Indeed, a woman described her efforts to change her obstetrician’s negative attitude in order to ‘take her seriously’ by invoking her professional expertise that allowed her to claim sophistication in the interpretation of readings. The quote below also reveals the user’s conviction that the doctor’s reservation may lie in the possibility of her becoming unnecessarily over-concerned, which in turn might increase the visits to the doctor.

But I thought, well, hey, you know, I’m going to tell him because…it’s all part of my management and then hopefully he would take me seriously. But I did feel that, because of his attitude, I had to explain to him that…I’d worked with a clinical background, and therefore I’d got the knowledge to be able to interpret the results intelligently. I wasn’t going to come to him with one elevated reading, that I would take multiple ones over a couple of days before I then came to see him. (F, 46, 1001)

A second explanation that was offered for doctors’ reservations was the belief that the traditional role of doctor might be questioned or threatened when the patient is trying to acquire more knowledge, to adopt an active role, and to assume more responsibility for health.

Because sometimes they feel, doctors, that…those people, if they are discussing with some [people] medical issues and they know that they are clever – they feel that we are getting cleverer than them. But actually, we would like – I told her I just like…I would like to understand the situation. It is good to educate yourself, and to [read] and to understand. (F, 33, 1017)

Although some participants described the tension that can be created in the patient-clinician relationship, others saw the practice of HBPM as a means to strengthen the relationship and to improve communication. Self-monitoring enabled users to convey concrete evidence of their condition to the doctor, rather than generalised and ambiguous descriptions, which in turn was expected to have a positive effect by increasing the doctor’s attention to the patient’s account.

I think the doctor will observe that you’re being a bit more serious than vaguely talking about this, that and the other. You go to the doctor and you have a pain and you’re vague about it, but if in fact you have…you’re serious enough to have done this, then I think they’ll listen to you, I think. (F, 93, 1012)

## Discussion

This study sought to examine the lay reasoning behind the interpretation of home BP readings and the communication of the practice of home monitoring to the doctor, among people who had instigated the purchase of the device themselves. Interpreting home BP readings involved a multifaceted reasoning that was based on lay models around the BP function, on rules of thumb that dictated the range of acceptable values, and on contextualising readings to personal contingencies. When readings were judged as problematic, people actively sought to identify the potential causes in order to decide on subsequent action. Actions were mostly related to instant lifestyle modifications while contacting the doctor was not initiated unless the problematic readings persisted, were not easily justified, or when the actions already taken were unsuccessful in regulating BP. Most participants had notified their doctor about the practice of HBPM, but not all experienced support. Participants’ lack of medical knowledge and the threat that doctors might feel when patients adopt an active role in their care were thought to account for doctors’ reservations. Nevertheless, HBPM was also seen as a means that can reinforce the patient-clinician relationship by facilitating communication within consultations.

Our findings are in line with the literature showing that people notice BP variability [14-17], associate its function with lifestyle factors [14,16] and initiate short-term lifestyle modifications [24] accordingly. Unlike previous research that shows that home readings are trusted when the device and the training to use it are provided by healthcare professionals [14,17] our findings indicate that BP fluctuations sometimes create confusion and uncertainty. This is in line with the results of research among hypertensive patients who self-initiate HBPM [16] and suggests that there is value in doctors engaging in discussions with their patients about these issues. Extending beyond previous research, our study identifies the rules of thumb that people use to judge whether a reading is normal. The BP values that are defined to be normal for adult populations is one such heuristic that constitutes a broad framework for assessment. This is supplemented by the, arguably more meaningful, personalised norms which are developed in accordance with the history of people’s BP and establish acceptable ranges of numbers. Moreover, the present evidence suggests that anchoring home readings to personal, daily-life circumstances is also important for sense-making when it comes to a reading.

This study is also in line with existing literature [16,17] in its observations about the varying degree of medical involvement. Participants narrated different levels of engagement by doctors, whilst some reported disinterest [24] or even reservations. This study further identifies the explanations that people provide for the perceived doctors’ disinterest or reservations. These appeal to users’ lack of medical knowledge and subsequent doctor’s distrust in their self-testing practices, and the possibility of doctors feeling threatened when patients adopt a more active stance in health management. On the one hand, using a home BP monitor saw the emergence of a patient-centred model where self-caring practices were a constituent element. On the other, the provision of ‘objective’ information about the patient condition allowed the strengthening of the patient-clinician relation and the alignment of competencies.

### Strengths and limitations

The qualitative nature of this study enabled a rich and detailed understanding of the reasoning lying behind the interpretation of home BP readings providing useful insights. Given that there is an increase in self-testing practices [25], it is vital to appreciate the lay reasoning behind the sense-making of results in order to fully realise the impact on self-care. Moreover, this study has linked the interpretation of readings with the relationship to the doctor illustrating the range of experiences that HBPM was viewed to have on patient-clinician communication.

Our participants were well educated and most had self-initiated the purchase and use of the monitor. These parameters may account for the good level of awareness of the guideline BP targets that the interviewees demonstrated, as they were probably more motivated to seek, and able to comprehend, relevant medical information. This is in line with research showing that those who engage with self-caring practices are more likely to be of an educated and/or affluent background [26]. Although the more educated composition of our sample may at first sight appear to undermine the value of the present findings, it is striking that the participants from this well-educated group still relied heavily on their personalised norms for assessing value acceptability.

### Implications for research or practice

These results show the importance of setting the context that would legitimise HBPM as part of self-care and that would make users feel confident to talk about this within consultations. This study suggests that patient confidence to the practice and trust to the readings would increase if there were discussions within consultations about the nature of BP and its inherent variability. Clear advice about the targeted BP values, the need to record them systematically and the conditions under which home measurements should be performed is important. Healthcare practitioners should also explore whether patients apply any personalised norm when judging value normality and whether this norm is appropriate from a clinical perspective. There was no indication in these data that contacting the doctor was a first line strategy when users encountered problematic readings. To the contrary, people had a conservative stance, and resorted to this when all other strategies to regulate BP failed. Examining healthcare professionals’ views on HBPM and other self-testing practices, and most importantly any concerns they might have, would significantly complement these findings.

## Conclusions

Medical technology for home use increasingly offers a wide range of devices that are simple to use. In tandem with this, as the prevailing discourse around health emphasises the role of individual autonomy and responsibility, lay engagement with self-caring practices is likely to further increase. By interviewing users of home BP monitors, we illustrated the reasoning implicated in the interpretation of home readings revealing the complexity but also the uncertainties that often characterise it. Greater medical involvement could assist in enabling people to acquire confidence in interpreting the readings and translating these into appropriate action. In turn, self-monitoring practices can afford opportunities to enhance communication between the patient and clinician.

## Abbreviations

HBPM: Home blood pressure monitoring; BP: Blood pressure.

## Competing interests

The authors declare that they have no competing interests.

## Authors’ contributions

KV contributed to the conception and design of the study, obtained ethical approval, collected, analysed and interpreted the data and wrote the manuscript. JB contributed to the conception and design of the study and to the analysis and interpretation of the data. TY contributed to the conception and design of the study. All authors critically revised and approved the final manuscript.

## Pre-publication history

The pre-publication history for this paper can be accessed here:

http://www.biomedcentral.com/1471-2296/14/97/prepub

## Supplementary Material

Additional file 1 Screening Questionnaire. Click here for file

Additional file 2 Interview Protocol. Click here for file
