# Supplementary material for: Interpreting and acting upon home blood pressure readings: a qualitative study
Source: BMC Fam Pract. 2013 Jul 13;14:97. doi: 10.1186/1471-2296-14-97 (PMC3726339; doi:10.1186/1471-2296-14-97)
Supplement: Additional file 1 — Screening Questionnaire. [file 1471-2296-14-97-S1.doc]

**Home Blood Pressure Monitors Questionnaire**

**The aim of this questionnaire is to gather some basic information about your home blood pressure monitor. There are also a few questions about you. This information will help me to confirm your eligibility to take part in this interview study. At the end of this questionnaire, you will be asked to confirm that you would be interested in taking part in an interview about how you use your blood pressure monitor. We ask you to provide your contact details so that if you are eligible and if you would like to take part, the researcher can contact you to arrange a convenient time. Please, note that all this information will be held and processed in the strictest confidence and in accordance with the Data Protection Act (1998). Only the researcher conducting this study will know your name. Other people involved in this project will not have access to your personal information.**

**Section 1: Your home blood pressure monitor**

1. Do you own a home blood pressure monitor? Yes No

2. Did you buy your home blood pressure monitor yourself? Yes No

**If Yes** - please go to question 5

3. If you have not personally purchased your home blood pressure monitor,

did another person (e.g. relative, friend) make that purchase on your behalf? Yes No

4. If your answers in questions 2 and 3 were “NO”, how did you come to own your home blood pressure monitor?

Please, briefly explain here …………………………………………………………………………………………………………………………..................

5. When did you buy your home blood pressure monitor? Month Year

6. In general, how often do you use your home blood pressure monitor?

At least once a day

A few times per week (please specify how many times…………………………………………………………………………)

About once a week

About once every two weeks

About once a month

Once every two months

Other (please specify………………………………………………………………………………………………………………………….)

7. What type of home blood pressure monitor do you have?

Automatic (or digital, these monitors have a display screen that shows the results in numerical forms)

Manual (or aneroid, these monitors include arm cuff, a squeeze bulb for inflation, a stethoscope or

microphone, and a medical gauge to measure the blood pressure. It is the type of monitor that most

doctors use)

I do not know what type of blood pressure monitor it is

8. Is your home blood pressure monitor validated? (Being validated means that it has gone through a series of tests to make sure it gives accurate and reliable results)

Yes

No

I do not know

9. What is the brand of your home blood pressure monitor?

Please specify here……………………………………………………………………………………………………………………………………………

10. What was the cost of your home blood pressure monitor?

Less than [£](http://en.wikipedia.org/wiki/Pound_sign)50

Between [£](http://en.wikipedia.org/wiki/Pound_sign)50 and [£](http://en.wikipedia.org/wiki/Pound_sign)100

More than [£](http://en.wikipedia.org/wiki/Pound_sign)100

11. What would you say was the main reason for purchasing your home blood pressure monitor?

Please specify here……………………………………………………………………………………………………………………………………………

……………………………………………………………………………………………………………………………………………………………………….

……………………………………………………………………………………………………………………………………………………………………….

12. Has your healthcare professional (e.g. doctor, nurse) ever noticed Yes No

any problem with your blood pressure?

13. What was that?

Please specify here……………………………………………………………………………………………………………………………………………

………………………………………………………………………………………………………………………………………………………………………

**Section 2: Your background information**

1. Gender

Female

Male

2. My age in years is:

3. Currently I am:

Employed

Unemployed

Retired

Other (Please specify…………………………………………………………………………….)

4. My nationality is (e.g. British, French): …………………………………………………………………………………

5. What is your highest educational qualification?

Degree or degree equivalent and above

Higher education to less than degree level (e.g. HND)

A level/Scottish Higher/Vocational level 3 and equivalent

O level/GCSE/Vocational level 2 and equivalent

No qualifications

6. What is your marital status?

Single

Married

Divorced

|  |
| --- |

**Section 3: My contact details**

As mentioned earlier, this questionnaire will help me make sure that the people who will be invited to take part in this research are those that are eligible and that would like be interviewed. In this last section, you are asked to provide your contact details so that the researcher can get in touch with you.

**Consent**

I consent to my personal details being held by the researcher conducting this study so as she will be able to contact me for inviting me to take part in the second stage of this research, which consists of an interview discussion.

At this stage I am just saying that I would like to receive more details, **I am NOT consenting to take part in the interview** and I understand that I am under no obligation to do this if I am invited to do so.

I understand that all personal data relating to research participants is held and processed in the strictest confidence, and in accordance with the Data Protection Act (1998).

Signed ______________________________ Date ____________________________________________

Name (block capitals please) _____________________________________________________________

Address_____________________________________________________________________________

____________________________________________________________________________________

____________________________________________________________________________________

Contact phone number _________________________________________________________________

Best time to contact____________________________________________________________________

Thank you very much for your time!
