# Supplementary material for: Interpreting and acting upon home blood pressure readings: a qualitative study
Source: BMC Fam Pract. 2013 Jul 13;14:97. doi: 10.1186/1471-2296-14-97 (PMC3726339; doi:10.1186/1471-2296-14-97)
Supplement: Additional file 2 — Interview Protocol. [file 1471-2296-14-97-S2.doc]

**Interview Protocol**

**Section A:** Issues around the purchase and the ownership of the home blood pressure monitor (HBPM) were examined in this section.

- Please, tell me about why you decided to buy a home blood pressure monitor.
- How long ago did you buy your HBPM?
- Did you have any reservations about buying a HBPM, at the time?
- Thinking back at the time, do you remember in what ways you had searched for your device?
- What were the factors that you took into account in order to make your purchase?
- If I asked you to think about your HBPM, what do you like about it?
- What don’t you like about your HBPM?
- Do you have any difficulty when you use it? Or is it generally easy in use?
- If a designer asked you, as a user of this device, to recommend any changes in the features of your HBPM, what would be those changes?

**Section B:** Issues around the storage of the device within the home environment and carriage outside this were explored in this section.

- Are there any times that you take your HBPM with you, outside the home?
- Where do you usually keep or store you HBPM within your home?

**Section C:** Patterns of device use were examined in this section.

- How often do you use this machine and why?
- Is there any particular time of the day?
- Are there times that you may forget to measure your blood pressure?
- How many readings are you taking every time you measure your blood pressure?
- Do you keep a record of your readings?
- Is it ever inconvenient for you to measure your BP?

**Section D:** In this section it was investigated whether participants discussed with other people that they owned and were using a HBPM.

- Do other people know that you measure your blood pressure? For example, your friends or family?
- Is it important for you that other people know / do not know and why?

**Section E.** The implications of home monitoring on the relationship with healthcare professionals were examined in this section.

- Would you say that the practice of measuring your blood pressure at home has changed in any way your relationship with your doctor? If yes, in what ways?
- Have you discussed with your doctor that you are measuring your blood pressure?
- If not, why?
- Do you generally discuss your readings with your doctor?

**Section F.** The way people made sense of home readings and the actions they were taking as a result of this were explored here.

- How and when do you know that your blood pressure is normal?
- When would you be concerned about the readings you are getting?
- What do you usually do when your readings are not so good?
- How do you know that your readings are trustworthy?

**Section G.** The potential impact of home monitoring on images of self and on health management was explored here.

- More generally, would you say that having this machine and measuring your blood pressure, has changed the way you feel about yourself or your health?
- If yes, in what ways?
